# Antibiotic self-medication in Otuke District, Northern Uganda: Prevalence and associated factors

**DOI:** 10.1371/journal.pone.0329290

**Published:** 2025-08-07

**Authors:** Denis Diko Adoko, Rebecca Nakaziba

**Affiliations:** 1 Department of Midwifery, Lira University, Lira, Uganda; 2 Department of Pharmacology, Lira University, Lira, Uganda; Children's National Hospital, George Washington University, UNITED STATES OF AMERICA

## Abstract

Antibiotic self-medication is a growing public health concern, particularly in low- and middle-income countries where access to healthcare is limited. The practice contributes to antimicrobial resistance which increases health care costs, morbidity, and mortality in the population. This study aimed to investigate the prevalence of antibiotic self-medication and its associated factors in the Otuke District, Northern Uganda. A community-based cross-sectional study was conducted in Otuke. Data was collected among adults aged 18 years and above using semi-structured questionnaire. Collected data was coded and double-entered into SPSS Software version 26 and exported to STATA 14 for analysis of frequencies and percentages. Modified Poisson regression was used to run analysis of the association at a P-value of 0.05. Out of the 385 participants, 261 (67.79%) reported having self-medicated with antibiotics in the past six months. The most commonly self-medicated antibiotics were amoxicillin 134 (51.3%), ampiclox 87 (33.3%) and metronidazole 57 (21.9%). Participants with previous successful treatment were 2.33 times more likely to self-medicate (PR = 2.33, 95% CI: 1.89–2.87, P < 0.001) while poor staff attitude increased the likelihood by 1.53 times (PR = 1.53, 95% CI: 1.38–1.71, P < 0.001). Knowledge about antibiotics was negatively associated with ASM in that those who had knowledge on antibiotics were 25% less likely to self-medicate with antibiotics (PR = 0.75, 95% CI: 0.65–0.86, P < 0.001). The practice of antibiotic self-medication was highly prevalent in Otuke district due to previous successful treatments and poor health care systems. The commonly self-medicated antibiotics were amoxicillin, ampiclox and metronidazole. We recommend public health interventions such as community education on antimicrobial resistance regulation of antibiotic use in the country.

## 1. Introduction

Antimicrobial self-medication (ASM) is a form of irrational drug use contributing to antimicrobial resistance, increased health care costs coupled with morbidity and mortality in the population globally [1,2]. It is a common form of healthcare practiced in most parts of the world, with over 50% of antibiotics bought and used over the counter [3]. In developed countries such as Saudi Arabia, antibiotics are prescription only drugs [4–6]. Unfortunately in developing countries, medicines use including antibiotics, is rarely regulated [7,8] leading to more cases of antibiotic resistance [9]. The prevalence of ASM in developed countries ranges from 3% to 19%, while that in developing countries ranges from 24% to 73.9% [10]. In Africa alone, the prevalence of ASM ranges from 12.1% to 93.9%, with a mean prevalence of 55.7% [11]. West Africa stands at a prevalence of 70.1%, whereas North Africa at 48.1% [11]. Studies conducted in Eritrea, Ghana, and Sudan depicted the prevalence of ASM at 45.1%, 36%, 71.3% respectively [12–14]. In Uganda, the prevalence of ASM was as high as 75.7% in 2014 and 93.8% among medical students in 2023 [15,16]. The main determinants of ASM include education level, age, gender, past successful use, severity of illness and income [3]. The most commonly self-medicated antibiotics are penicillins, metronidazole, and ceftriaxone in Afghanistan [17]; ciprofloxacin in Pakistan [18]; and amoxicillin, metronidazole [19], and Co-trimoxazole [16] in Uganda. While the development of antibiotics was one of the great discoveries of modern medicine [20], improper use of antibiotics has led to an increasing number of bacteria becoming resistant to these drugs [21]. Antimicrobial resistance (AMR) has been a global public health concern since the past decade [22] with the number of antibiotic-resistant microorganisms rapidly on the rise [23]. Although AMR is a natural phenomenon, resistance develops more rapidly through the misuse and overuse of antimicrobial agents [24,25]. Thus antibiotic prescriptions are especially important for vulnerable populations, such as immunocompromised patients, as they help prevent the development of drug resistance and the emergence of novel bacterial phenotypes within these groups [26].

Although the government of Ugandan has policies and laws against antibiotic use [27,28], they are not effectively enforced. As such, antibiotics can be accessed without a prescription from community drug shops and pharmacies [29]. Otuke District in particular hosts about 50 drug shops, a situation that facilitates easy over-the-counter access to antibiotics without a prescription. Despite this widespread availability, there is a significant lack of data on the extent and patterns of antibiotic self-medication in Otuke District. This gap in evidence presents a major barrier to informed decision-making in the district. Therefore, there was a pressing need for strong, well-designed research to generate reliable data on community antibiotic use in Otuke District. Such evidence is critical to guide the planning and implementation of targeted interventions aimed at curbing non-prescription antibiotic use and mitigating its public health consequences.

The current study aimed to determine the prevalence and practices of antibiotic self-medication in Otuke District, Northern Uganda, as well as the associated factors in order to inform concerned stakeholders of the vice so that informed interventions can be designed. The study found a high rate of antibiotic self-medication in the area due poor health systems.

## 2. Materials and methods

### 2.1. Study area

The study was performed in the Otuke District located within the Lango sub-region in Northern Uganda. Otuke district consists of many government health facilities that are well distributed across all sub-counties and a total of 50 drug shops. The Otuke Town Council alone has five medical clinics (*Source: Records from the National Drug Authority, Otuke District).*

### 2.2. Participant recruitment and procedures

The study was a descriptive cross-sectional study employing quantitative methods of data collection. A cross-sectional design is time-saving and inexpensive. The sample size was calculated using the Kish Leslie (1965) formula assuming a proportion of 50% and yielded a sample size of 385. The study was conducted among adult community members (aged 18 years and above) of the Otuke District from the 27^th^ of March to 20^th^ April, 2023 excluding those who were very sick and could not respond to the questions. The study employed a cluster sampling technique. The clusters were randomly selected from the Adwon-Ibuto, Barodugu, Teogini and Te-Boke cells of the Otuke Town Council. The study participants were selected as per the convenient sampling technique by interviewing readily available participants from each cluster. The potential participants were accessed by the research team moving into their homes. The study purpose, risks and benefits were clearly explained to potential participants who were required to sign an informed consent form to affirm their acceptance. Those who agreed to participate in the study and signed the consent form were recruited. The data were collected using a pretested questionnaire developed in line with the study objectives. The tool had three parts: Part A (Sociodemographic characteristics such as age, sex, and level of education), Part B (Self-medication practices), and Part C (Factors associated with ASM (Health system factors, individual factors, and interpersonal factors). Following the acquisition of informed consent, data were collected using an interviewer-administered questionnaire. Each section of the questionnaire was well explained to the respondents who then provided answers. The questionnaires were checked for completeness after data collection, and those with incomplete or irrelevant data were excluded from the analysis. The collected data were coded and double-entered into Statistical Package for Social Sciences (SPSS) version 26 to ensure accuracy. Thereafter, the dataset was exported to Stata version 14 for statistical analysis. Descriptive statistics were conducted to summarize the data, with frequencies and percentages presented in tables and figures for categorical variables.

To assess associations between independent variables and antibiotic self-medication (ASM), both bivariate and multivariate analyses were conducted using modified Poisson regression with robust error variance. This method was preferred to estimate prevalence ratios (PRs) due to the cross-sectional design and the relatively high prevalence of the outcome.

Variables with a P-value ≤ 0.05 at 95% confidence intervals in the multivariate model were considered statistically significant and thus associated with ASM. The multivariate analysis also served to control for potential confounding variables.

### 2.3. Ethical issues

The study was presented before the faculty of Nursing and Midwifery, Lira University and to Otuke district administration for approval (LUFM-2023–001). Moreover, the study purpose, risks and benefits were clearly explained to potential participants (adults aged 18 years and above) who were required to sign an informed consent form to affirm their acceptance before recruitment into the study.

## 3. Results

### 3.1. Socio-demographic data

Among the 385 participants, 94.81% were of the Lango ethnic group, 49.35% were aged 18–28 years, 56.62% were females, 54.55% had attained primary education, 65.45% were self-employed (Table 1).

**Table 1:** Socio-demographic characteristics of the study participants.

Variable		Frequency	Percent
**Age (years)**	18–28	190	49.35
	29–40	114	29.61
	41–60	64	16.62
	61 and above	17	4.42
**Gender**	Male	167	43.38
	Female	218	56.62
**Educational level**			
No formal education	No	377	97.92
	Yes	8	2.08
Primary education	No	175	45.45
	Yes	210	54.55
Secondary education	No	264	68.57
	Yes	121	31.43
Tertiary education	No	337	87.53
	Yes	48	12.47
**Marital status**	Single	179	46.49
	Married	206	53.51
**Occupation**	Not employed	63	16.36
	Self-employed	252	65.45
	Not employed	63	16.36
	Government employee	40	10.39
	Private sector	30	7.79
**Tribe**	Lango	365	94.81
	Others	20	5.19
**Religion**	Christians	375	97.4
	Others	10	2.6

### 3.2. Prevalence of antibiotic self-medication

**Fig 1:**
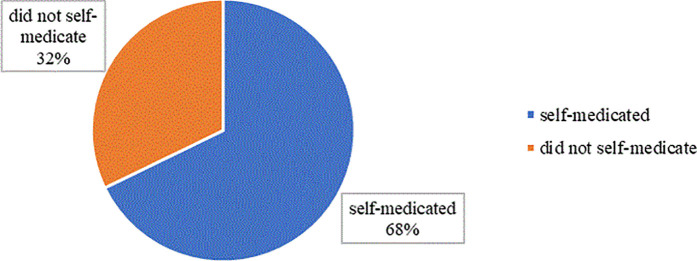
Prevalence of antibiotic self-medication in Otuke district (n = 385).

The majority of the community members of the Otuke Town Council (67.79%) practiced self-medication (Fig 1)

### 3.3. Commonly self-medicated antibiotics and their sources

The most common self-medicated antibiotic was amoxicillin (51.3%). Most of the respondents (98.5%) obtained antibiotics from drug shops/pharmacy (Table 2).

**Table 2:** Commonly self-medicated antibiotics and their sources.

Variable	Number (n)	Percentage (%)
***Self- medicated antibiotics***		
Amoxicillin	134	51.3
Ampiclox	87	33.3
Cloxacillin	24	9.2
Metronidazole	57	21.9
Erythromycin	28	10.8
Azithromycin	13	5.0
Cotrimoxazole	21	8.1
Ciprofloxacin	42	16.1
Doxycycline	24	9.2
Other antibiotics	35	13.5
***Sources of antibiotics for self-medication***		
Drug shop/pharmacy	257	98.5
Friends/relatives	3	1.1
Previous remainder	1	0.4
Others	00	0.0

### 3.4. Factors associated with antibiotic self-medication

#### 3.4.1. Health system, individual and interpersonal factors.

Among the health system factors, individual factors and interpersonal factors; long waiting hours (82.9%), previous successful treatment (55.6%), and suggestions by friends/relatives (24.4%) had the greatest effects respectively (Table 3).

**Table 3:** Health system, individual and interpersonal factors associated with ASM.

Variable	Number (n)	Percentage (%)
**Health system factors**		
Poor staff attitude	110	28.6
Health facility too far	55	14.3
Long waiting hours	319	82.9
No drugs at the health facility	268	69.9
Lack of medical professional	35	9.1
Unregulated distribution of antibiotics	89	23.1
**Individual factors**		
Previous symptom experience	207	53.8
Previous successful treatment	214	55.6
Mild Illness	211	54.8
Storage of antibiotics	109	28.3
Lack of time to visit doctors	70	18.2
Negative attitude towards health workers	21	5.5
Inability to pay for the health charges	48	12.5
Freedom of choice	177	46.1
Knowledge on the antibiotics	157	40.8
Old prescription	113	29.4
**Interpersonal factors**		
Given by a friend	43	11.2
Suggestion by a relative/friend	94	24.4
Drug brought by health worker	41	10.6

#### 3.4.2. Associations between socio-demographic characteristics with ASM.

There were no significant associations between antibiotic self-medication and age, gender, marital status, occupation, tribe, or religion (all p > 0.05). Only tertiary education was significantly associated with self-medication (PR = 1.27, 95% CI: 1.10–1.47; p = 0.002), indicating those with tertiary education were 1.27 times more likely to self-medicate (Table 4).

**Table 4:** Association of socio-demographic characteristics with ASM (at 95% CI).

Variable	Category	PR (95% CI)	P-value
Age	18-28	Ref	
	29–40	1.05 (0.90–1.23)	0.542
	41–60	0.96 (0.78–1.18)	0.691
	61 and above	1.14 (0.86–1.52)	0.35
Gender	Male	Ref	
	Female	0.99 (0.86–1.13)	0.862
Level of education			
No formal education	No	Ref	
	Yes	1.30 (0.99–1.70)	0.059
Primary	No	Ref	
	Yes	0.92 (0.80–1.06)	0.238
Secondary	No	Ref	
	Yes	0.93 (0.80–1.09)	0.358
Tertiary	No	Ref	
	Yes	1.27 (1.10–1.47)	0.002*
Marital status	Single	Ref	
	Married	1.01 (0.88–1.15)	0.939
Occupation			
Not employed	No	Ref	
	Yes	1.11 (0.94–1.31)	0.216
Self-employed	No	Ref	
	Yes	0.89 (0.77–1.02)	0.09
Gov’t employee	No	Ref	
	Yes	1.10 (0.89–1.35)	0.372
Private sector	No	Ref	
	Yes	1.00 (0.77–1.30)	0.997
Tribe	Others	Ref	
	Lango	1.11 (0.86–1.45)	0.426
Religion	Others	Ref	
	Christianity	1.19 (0.86–1.63)	0.294

#### 3.4.3. Associations between health system factors with ASM.

Bivariate analysis showed that participants who reported that the health facility was too far were 1.6 times more likely to self-medicate with antibiotics compared to those who did not (PR = 1.60, 95% CI: 1.47–1.74, p < 0.001). Similarly, those who experienced poor staff attitude were also 1.6 times more likely to self-medicate (PR = 1.60, 95% CI: 1.43–1.80, p < 0.001). Participants who perceived inadequate medical personnel were 1.55 times more likely to self-medicate (PR = 1.55, 95% CI: 1.43–1.67, p < 0.001). However, reporting lack of drugs at the facility was not significantly associated with self-medication (PR = 1.01, 95% CI: 0.87–1.17, p = 0.94) (Table 5).

**Table 5:** Association of health system factors with ASM.

Variable		PR (95% CI)	P-value
Health facility too far	No	Ref	
	Yes	1.60 (1.47–1.74)	<0.001*
Poor staff attitude	No	Ref	
	Yes	1.60 (1.43–1.80)	<0.001*
No drugs at the facility	No	Ref	
	Yes	1.01 (0.87–1.17)	0.94
Inadequate medical personnel	No	Ref	
	Yes	1.55 (1.43–1.67)	<0.001*

#### 3.4.4. Associations between interpersonal factors with ASM.

Bivariate analysis revealed strong associations between interpersonal influences and antibiotic self-medication. Participants who received antibiotics from a friend were 1.57 times more likely to self-medicate (PR = 1.57, 95% CI: 1.45–1.70, p < 0.001). Those who acted on a suggestion by a relative were 1.71 times more likely to self-medicate (PR = 1.71, 95% CI: 1.55–1.90, p < 0.001). Similarly, participants who were given antibiotics by a health worker informally were 1.56 times more likely to engage in self-medication (PR = 1.56, 95% CI: 1.44–1.69, p < 0.001) (Table 6).

**Table 6:** Association between interpersonal factors with ASM.

Variable		PR (95% CI)	P-value
Given by a friend	No	Ref	
	Yes	1.57 (1.45–1.70)	<0.001*
Suggestion by a relative	No	Ref	
	Yes	1.71 (1.55–1.90)	<0.001*
Drug brought by health worker informally	No	Ref	
	Yes	1.56 (1.44–1.69)	<0.001*

#### 3.4.5. Associations of individual factors with ASM.

The bivariate analysis revealed that all individual factors were significantly associated with ASM (p < 0.001). Participants who had previously treated themselves successfully with antibiotics were 3.06 times more likely to self-medicate (PR = 3.06, 95% CI: 2.45–3.83, p < 0.001). Those who perceived their illness as not serious were 1.93 times more likely to engage in ASM (PR = 1.93, 95% CI: 1.63–2.30, p < 0.001), while individuals who stored antibiotics for future use had a 1.82 times higher likelihood (PR = 1.82, 95% CI: 1.63–2.02, p < 0.001). Lack time to visit doctors (PR = 1.65, 95% CI: 1.51–1.80, p < 0.001), misconceptions about health workers (PR = 1.52, 95% CI: 1.41–1.63, p < 0.001), inability to pay for medical services (PR = 1.58, 95% CI: 1.46–1.72, p < 0.001), and the use of old prescriptions (PR = 1.84, 95% CI: 1.65–2.05, p < 0.001) were also significantly associated with increased likelihood of ASM (Table 7).

**Table 7:** Association of individual factors with ASM.

Variable		PR (95% CI)	P-value
Previous successful treatment	No	Ref	
	Yes	3.06 (2.45–3.83)	<0.001*
Illness was not serious	No	Ref	
	Yes	1.93 (1.63–2.30)	<0.001*
Store antibiotics for future use	No	Ref	
	Yes	1.82 (1.63–2.02)	<0.001*
Lack time to visit doctors	No	Ref	
	Yes	1.65 (1.51–1.80)	<0.001*
Misconception on health workers	No	Ref	
	Yes	1.52 (1.41–1.63)	<0.001*
Inability to pay for health charges	No	Ref	
	Yes	1.58 (1.46–1.72)	<0.001*
Old prescription	No	Ref	
	Yes	1.84 (1.65–2.05)	<0.001*

### 3.5. Multivariate analysis of the association of factors with ASM

Multivariate analysis was done by running all variables with P ≤ 0.2 at bivariate level. Several factors were identified that remained significantly associated with antibiotic self-medication (ASM) after adjusting for confounders. Thirteen factors were significantly associated with antibiotic self-medication (ASM). Participants with tertiary education were 1.23 times more likely to self-medicate compared to those without (PR = 1.23, 95% CI: 1.05–1.44, P = 0.011). Poor staff attitude increased the likelihood by 1.53 times (PR = 1.53, 95% CI: 1.38–1.71, P < 0.001), while those living far from health facilities were 1.45 times more likely to self-medicate (PR = 1.45, 95% CI: 1.32–1.60, P < 0.001). Inadequate medical personnel was associated with a 1.32-fold increase (PR = 1.32, 95% CI: 1.17–1.50, p < 0.001). Participants with previous successful treatment were 2.33 times more likely to self-medicate (PR = 2.33, 95% CI: 1.89–2.87, P < 0.001). Those perceiving their illness as not serious had a 1.26 times higher likelihood (PR = 1.26, 95% CI: 1.08–1.46, P = 0.003). Lack of time to visit doctors increased self-medication by 1.29 times (PR = 1.29, 95% CI: 1.16–1.44, p < 0.001), while misconceptions about health workers raised it by 1.38 times (PR = 1.38, 95% CI: 1.12–1.71, p = 0.003). Inability to pay medical charges was associated with a 1.35-fold increase (PR = 1.35, 95% CI: 1.18–1.56, p < 0.001). Use of old prescriptions increased the likelihood by 1.58 times (PR = 1.58, 95% CI: 1.43–1.75, p < 0.001). Being influenced by relatives raised the likelihood by 1.25 times (PR = 1.25, 95% CI: 1.13–1.38, p < 0.001), and drugs brought by health workers were associated with a 1.24 times higher likelihood of self-medication (PR = 1.24, 95% CI: 1.08–1.43, p = 0.003). Knowledge on antibiotics was negatively associated with ASM in that those who had knowledge on antibiotics were 25% less likely to self-medicate with antibiotics (PR = 0.75, 95% CI: 0.65–0.86, P < 0.001).

Factors like no formal education, storing antibiotics, being self-employed, or being given drugs by a friend were not significantly associated with ASM (Table 8).

**Table 8:** Multivariate analysis of factors associated with antibiotic self-medication.

Variable		Bivariate analysis		Multivariate analysis	
		**PR (95% CI)**	**P-value**	**aPR (95% CI)**	**P-value**
No formal education	No	Ref		Ref	
	Yes	1.30 (0.99–1.70)	0.059	1.19 (0.90–1.58)	0.225
Tertiary	No	Ref		Ref	
	Yes	1.27 (1.10–1.47)	0.002*	1.23 (1.05–1.44)	0.011*
Self-employed	No	Ref		Ref	
	Yes	0.89 (0.77–1.02)	0.09	0.94 (0.82–1.07)	0.335
Poor staff attitude	No	Ref		Ref	
	Yes	1.60 (1.43–1.80)	<0.001*	1.53 (1.38–1.71)	<0.001*
Health facility too far	No	Ref		Ref	
	Yes	1.60 (1.47–1.74)	<0.001*	1.45 (1.32–1.60)	<0.001*
Inadequate medical personnel	No	Ref		Ref	
	Yes	1.55 (1.43–1.67)	<0.001*	1.32 (1.17–1.50)	<0.001*
Previous successful treatment	No	Ref		Ref	
	Yes	3.06 (2.45–3.83)	<0.001*	2.33 (1.89–2.87)	<0.001*
Illness not serious	No	Ref		Ref	
	Yes	1.93 (1.63–2.30)	<0.001*	1.26 (1.08–1.46)	0.003*
Knowledge on antibiotics	No				
	Yes	0.53 (0.44–0.64)	<0.001	0.75 (0.65–0.86)	<0.001
Store antibiotics for future	No	Ref		Ref	
	Yes	1.82 (1.63–2.02)	<0.001*	0.94 (0.85–1.04)	0.255
inadequate time to visit doctors	No	Ref		Ref	
	Yes	1.65 (1.51–1.80)	<0.001*	1.29 (1.16–1.44)	<0.001*
Misconception on health workers	No	Ref		Ref	
	Yes	1.52 (1.41–1.63)	<0.001*	1.38 (1.12–1.71)	0.003*
Inability to pay charges	No	Ref		Ref	
	Yes	1.58 (1.46–1.72)	<0.001*	1.35 (1.18–1.56)	<0.001*
Old prescription	No	Ref		Ref	
	Yes	1.84 (1.65–2.05)	<0.001*	1.58 (1.43–1.75)	<0.001*
Given by a friend	No	Ref		Ref	
	Yes	1.57 (1.45–1.70)	<0.001*	1.02 (0.91–1.14)	0.72
Suggestion by a relative	No	Ref		Ref	
	Yes	1.71 (1.55–1.90)	<0.001*	1.25 (1.13–1.38)	<0.001*
Drug brought by health worker	No	Ref		Ref	
	Yes	1.56 (1.44–1.69)	<0.001*	1.24 (1.08–1.43)	0.003*

## 4. Discussion

### Summary of the findings

Out of the 385 participants, 261 (67.79%) reported having self-medicated with antibiotics in the past six months. The most commonly self-medicated antibiotic were amoxicillin 134 (51.3%), ampiclox 87 (33.3%), metronidazole 57 (21.9%), and ciprofloxacin 42 (16.1%).

Participants with tertiary education were more likely to self-medicate compared to those without. Poor staff attitude, living far from health facilities, and inadequate medical personnel each increased the likelihood of self-medication. Those with previous successful treatment were much more likely to self-medicate. Perceiving the illness as not serious, lacking time to visit doctors, and having misconceptions about health workers also raised the likelihood. Additionally, inability to pay medical charges, use of old prescriptions, influence by relatives, and drugs brought by health workers were all linked to higher chances of self-medication. Knowledge on antibiotics was negatively associated with ASM in that those who had knowledge on antibiotics were less likely to self-medicate with antibiotics. Factors such as no formal education, storing antibiotics, being self-employed, or being given drugs by a friend were not significantly associated with ASM.

### Prevalence of ASM

Of the 385 participants, 68% (over two-thirds) reported having self-medicated with antibiotics in the past 6 months. This implies that approximately 7 in 10 community members practiced ASM, with 49.4% of ASM being in the age bracket 18–28 years. This could be attributed to the fact that there are no regulations in place to curb the practice, so every member of the community is free to buy antibiotics from the many established drug shops or pharmacies. This could also be the result of the long waiting hours experienced when patients seek health care services in various health facilities. The findings of this study are closely related to those of a similar study in Sudan, where the prevalence of ASM was 71% [14]; and to another in northern Uganda, which was 73% [16]; and in Afghanistan, at 73.2% [17]. Against this background, a way forward is worth seeking to reduce the practice of ASM among the community. On the other hand, a similar study conducted among nursing students in Western Uganda showed a much greater prevalence of ASM, at 85.7% [19]. This could be due to the pharmacological knowledge gained by the nursing students. Comparatively, a similar finding of 73.2% was found in Afghanistan [17]. These similarities may be attributed to common behavioural tendencies such as reliance on past experiences with antibiotics, perceived convenience, and insufficient enforcement of prescription-only regulations. Additionally, the lack of awareness regarding antibiotic resistance in both urban and rural populations contributes to these trends. However, this prevalence is much lower compared to the 93.8% found among undergraduate students in Eastern Uganda [15], likely due to differences in population characteristics; -students are more educated, have easier access to pharmacies, and are more confident in self-diagnosing. In contrast, the general rural population in Otuke may have limited access and less exposure to self-medication practices. These contextual differences account for the observed variation. On the other hand, the prevalence in Otuke was higher than the 58% in Tanzania [30], 45.1% in Eritrea [12] and 40.4% reported in Jordan [31]. This was even much higher than the 31.7% observed in the United Arab Emirates [32], 10.32% in China [33], 36.1% to 45.8% Middle East and 29%, South America [10]. This could be due to better drug use regulations that guide antimicrobial use in these countries, whereby antibiotics are prescription only drugs. In Otuke, the rural context, limited health infrastructure, and frequent stockouts may compel individuals to resort to self-medication more often than those in urbanized or better-resourced areas.

### Antibiotics self-medicated and their sources

Of the 385 participants, 98.5% bought antibiotics from drug shops/pharmacies. This study also explored the common self-medicated antibiotics and discovered amoxicillin (51.3%), ampiclox (33.3%), and metronidazole (21.9%). In western Uganda among nursing students, amoxicillin and metronidazole were the most commonly used antibiotics [19] as well as in northern Uganda as reported by Ocan, Bwanga [16]. In Sudan, amoxicillin is the most commonly used antibiotic [14]. The most frequently used antibiotic for self-medication was amoxicillin (51.3%), followed by ampiclox (33.3%), metronidazole (21.9%), and ciprofloxacin (16.1%). This pattern is in agreement with findings from Saudi Arabia, where amoxicillin was also the most commonly used antibiotic [34], and Afghanistan, where overuse of broad-spectrum antibiotics like amoxicillin and ciprofloxacin was reported [17]. The widespread self-medication with amoxicillin may be due to its easy availability, relatively low cost, broad-spectrum activity, and the public’s perception that it is a “safe and effective” first-line treatment for many infections [17,33]. Moreover, since amoxicillin is commonly prescribed in public health facilities, people may become familiar with its use and reuse it without medical guidance. However, the reliance on these few antibiotics raises concerns about inappropriate usage and the growing threat of antimicrobial resistance (AMR), especially when full dosages are not completed or when used without confirmed diagnosis (Torres et al., 2019). The increased use of amoxicillin could be due to its lower cost than other antibiotics and the high prevalence of respiratory tract infections (although not all respiratory infections require antibiotics for treatment) [35]. Sensitizing the community to various treatment modalities for respiratory infections could be vital for minimizing ASM.

### Factors associated with ASM

A variety of factors were analyzed for their association with ASM in this study grouped into health system, individual and interpersonal factors. Participants with tertiary education were more likely to self-medicate compared to those with lower or no formal education. This finding aligns with results from China, where more educated individuals felt confident in diagnosing and treating themselves [33]. This behaviour might stem from a preconceived sense of medical knowledge or greater access to information via the internet. However, unlike expectations that education should reduce risky health behaviours, higher education may paradoxically increase self-medication if not paired with adequate health literacy on antimicrobial resistance. Structural issues such as poor attitude of health workers, long distance to health facilities, and inadequate medical personnel were significantly associated with higher self-medication. These findings are in line with the systematic review by Torres, Chibi [36], which showed that dissatisfaction with healthcare services often drives people to self-medicate. In Otuke, where health facilities are understaffed, frustration likely encourages patients to seek quick alternatives like pharmacies or leftover drugs at home. The study found that individuals who had previously used antibiotics successfully were much more likely to self-medicate. This is supported by findings in Saudi Arabia and Jordan [31,34] where prior positive experiences with antibiotics led individuals to repeat the behavior, regardless of the current illness. The confidence gained from previous success may overshadow concerns about correct dosage or diagnosis. Many participants reported not perceiving their illness as serious, having no time to visit health facilities, or holding misconceptions about health workers, all of which contributed to self-medication. Similar behavioral explanations were cited in studies from Tanzania [30] and Eritrea [12], where minor symptoms were viewed as unworthy of professional consultation. This perception lowers the threshold for engaging in self-medication. Other factors included lack of money to pay medical fees, use of old prescriptions, influence by friends or family, and direct access to drugs from health workers. These are common in resource-limited settings, as reported in Cameroon and other African countries [37]. Economic hardship often forces people to opt for cheaper, more accessible alternatives, even if unsafe. Additionally, social networks and informal sources of medication, such as leftover prescriptions or drugs sold by health workers, perpetuate the practice [36]. This finding is similar to that of Sachdev and others who found that unregulated deliveries were the major driver of ASM [38]. Therefore, the enactment of drug regulation laws could be the beginning of an end to ASM. Knowledge on antibiotics was negatively associated with ASM in that those who had knowledge on antibiotics were less likely to self-medicate with antibiotics. This finding re-echoes that of Ahmed, King [39] who discovered that people with greater knowledge of antibiotics were less likely to self-medicate.[39]. Previous symptom experience was also significantly associated with ASM probably because individuals tend to associate a given symptom with a disease. Therefore, a symptom managed successfully in the past is deemed manageable in the same way in the future. This is in agreement with findings of a research conducted in Pakistan [40]. Although many symptoms may be successfully managed by a specific drug, different conditions can manifest in a similar way, leading to the irrational use of antibiotics. Communities should be aware that some diseases mimic each other and that the use of a given antibiotic could be more of a disaster than a blessing. In addition, previous successful treatment was highly significantly associated with ASM. This could have been due to the patient’s ability to remember how he/she was managed. This finding is consistent with a study performed in low- and middle-income countries (LMICs) in which the habit of self-medication using previous treatments was identified [41]. There is a need for more patient and family sensitization to the dangers of practicing ASM. Furthermore, long waiting hours in health facilities were reported by multiple respondents in the present study were associated with ASM. Many respondents thought it was a waste of time going to a health facility at 8:00 am and returning home late in the afternoon. Therefore, they end up leaving the facility in search of quicker services at drug stores. In Ethiopia, a similar study supported the present findings where long waiting times contributed to self-medication [42]. Increased staffing could reduce the time spent at the facility during health seeking and improve patient trust. Moreover, poor staff attitude was another factor that was significantly associated with ASM in this study. Staff attitudes in terms of being rude, asking for money or not willing to help caused the community members to go elsewhere and buy their own drugs without consultation. This finding was consistent with those from studies in Cameroon and LMICs [36,43]. Regular staff motivation and appraisal could instill and sustain the drive to serve the medical staff, which could improve medication practices with antibiotics and prevent ASM.

## Conclusion and recommendations

There was a high prevalence of antibiotic self-medication in the Otuke District. The most common self-medicated antibiotics were broad-spectrum agents like amoxicillin, ampiclox, metronidazole, and ciprofloxacin. Key factors associated with ASM practice were tertiary education, poor staff attitude, lacking time to visit doctors, living far from health facilities, previous successful treatment and inadequate medical personnel.

The ministry of health should strengthen regulation and enforcement of prescription-only policies and scale up national awareness campaigns on antibiotic resistance

### Study strengths and limitations

This study provided valuable insights into the prevalence and drivers of antibiotic self-medication in a rural Ugandan setting, forming a basis for public health interventions and further research.

Despite this, several limitations should be considered when interpreting the findings:

Firstly, the study relied heavily on self-reported data, which may have introduced recall bias and social desirability bias. Participants may have either underreported or exaggerated their antibiotic use due to forgetfulness or the tendency to provide socially acceptable responses. Secondly, the use of a cross-sectional study design limited the ability to establish causal relationships between variables. The findings represent associations at a specific point in time and do not account for changes in behavior or exposures over time. Although the study design could not establish causality, efforts were made to strengthen internal validity by including a wide range of relevant variables and using appropriate statistical analyses (modified Poisson regression) to explore associations between factors and antibiotic self-medication.

Thirdly, there is a possibility of sampling bias. For instance, individuals who were not available during the data collection period such as working adults may have been underrepresented, potentially affecting the representativeness of the sample. To reduce sampling bias, data collection was scheduled at different times of the day and on both weekdays and weekends to ensure that both working and non-working individuals had an opportunity to participate. This helped include a more diverse range of respondents and minimized the risk of underrepresenting any specific subgroup.

Fourthly, the study did not incorporate clinical validation of antibiotic use. All responses were based on participants’ recall without verification through prescriptions or pharmacy records. This limited the accuracy of the information on the type and appropriateness of antibiotic use. Although clinical verification was not possible, participants were asked to name or describe the antibiotics they had used, and the data collection tool included a checklist of commonly used antibiotics in Uganda to help prompt accurate recall.

## Supporting information

S1 File PLOS Human Participants Research Checklist_R. (DOCX)
